# Compound heterozygosity for two GHR missense mutations in a patient affected by Laron Syndrome: a case report

**DOI:** 10.1186/s13052-017-0411-7

**Published:** 2017-10-12

**Authors:** Stefania Moia, Daniele Tessaris, Silvia Einaudi, Luisa de Sanctis, Gianni Bona, Simonetta Bellone, Flavia Prodam

**Affiliations:** 1 Division of Pediatrics, Department of Health Sciences, University of Piemonte Orientale, Novara, Italy; 2 Pediatric Endocrinology, Regina Margherita Children Hospital, University of Turin, Torino, Italy; 3 Division of Endocrinology, Department of Translational Medicine, University of Piemonte Orientale, Novara, Italy

**Keywords:** Growth Hormone, Growth Hormone Receptor, Laron Syndrome, Short Stature

## Abstract

**Background:**

Mutations localized in the Growth Hormone Receptor (GHR) gene are often associated with the pathogenesis of Laron Syndrome, an autosomal recessive hereditary disorder characterized by severe growth retardation. Biochemically, patients present normal to high circulating GH levels, in presence of very low or undetectable IGF-I levels, which do not rise after rhGH treatment.

**Case presentation:**

We describe the case of a 3.8 years old girl with symmetrical short stature (−3.76 SDS), low IGF-1 and IGFBP-3, in presence of normal GH levels. Parents were not relatives and there was no family history of short stature. During the second day of birth, she developed severe hypoglycaemia that required glucose infusion. She presented frontal bossing and depressed nasal bridge. IGF-1 generation test showed no response, suggesting a GH resistance evidence. In the hypothesis of Laron Syndrome, we decided to perform a molecular analysis of Growth Hormone Receptor (GHR) gene. This analysis demonstrated that the patient was compound heterozygote for two missense mutations.

**Conclusions:**

GHR gene mutations are a well demonstrated cause of GH insensitivity. In heterozygous patients, probably the normal stature may be achieved by a compensatory mechanism of GH secretion or signalling. On the contrary, in homozygous or compound heterozygous patients these compensatory mechanisms are inadequate, and short stature may be the consequence.

## Background

The Growth Hormone Receptor (GHR) is a type I transmembrane glycoprotein that belongs to the cytokine receptor superfamily [1]. The human gene maps to chromosome 5p13.1–p12 and is composed by 10 exons, with exon 1 and a large part of exon 10 that are untranslated regions and most of exon 2 that encodes for the signal peptide [2, 3]. The signal peptide (residues 1–18) is later removed from the mature protein that contains 638 amino acids [4]. In particular, exon 2–7 of GHR gene encoded for the extracellular domain of the receptor, involved in GH hormone binding, exon 8 for the transmembrane domain and exon 9–10 encoded for the cytoplasmic domain that activates the JAK-STAT signalling pathway [5, 6]. GHR is expressed as a dimer on the cell surface, with a single GH molecule that binds two GHRs extracellular domains sequentially and induces a conformational change in the receptor, essential for signal transduction [7].

Defects in the GHR protein, resulting from abnormalities of the GHR gene, have been shown to result in the clinical phenotypes of classical Growth Hormone Insensitivity or Laron Syndrome [5, 8]. The first is clinically characterized by severe postnatal growth failure and facial dysmorphism, and biochemically by normal-to-elevated circulating GH concentrations but deficiencies in circulating Insulin-like Growth Factor 1 (IGF-1) and Insulin-like Growth Factor Binding Protein 3 (IGFBP-3) [9]. Laron Syndrome is an autosomal recessive hereditary disorder characterized by severe growth retardation (height − 3 to −10 standard deviation score [SDS]), small cranium with underdeveloped facial bones, acromicria and organomicria, obesity and hypogenitalism. In addition, circulating GH levels are high in presence of low-to-undetectable IGF-I levels, which do not rise after short-term or long-term rhGH administration. The only treatment for Laron Syndrome is daily rhIGF-I administration [10].

In this report, we described a child referred to our Pediatric Endocrinology Department for a severe growth failure, in presence of low IGF-1 and IGFBP-3, but normal GH levels. After exclusion of a growth hormone deficiency condition, we performed direct sequencing of GHR gene sequence. This analysis demonstrated that the patient was compound heterozygote for two missense mutations. In particular, one of these mutations involved the first translation initiation codon of the protein. Consequently, the correct expression of the receptor will be most likely inhibited, probably causing the observed Laron phenotype.

## Case presentation

**Fig. 1:**
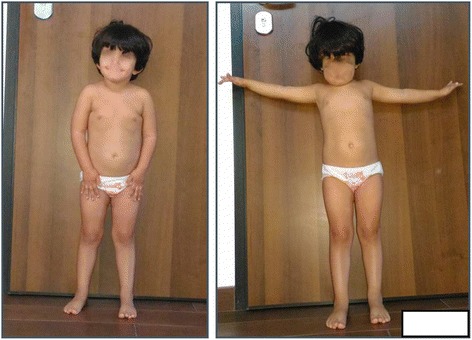
Physical presentation at 3.8 years old

We report a 3.8 years old girl who was referred to our Pediatric Endocrinology Department for short stature. At first evaluation she was 1.6 years old. She presented a symmetrical short stature: height was 72.5 cm (−2.58 SDS), sitting height was 46.1 cm (−2.3 SDS), sitting height/height ratio was 0.63 (0 SDS), weight was 8.2 kg (−3.15 SDS) and head circumference was 46.8 cm (+0.31 SDS). She was prepuberal. Facial features included frontal bossing and depressed nasal bridge. No other congenital abnormalities were evident (Fig. 1). Subscapular skinfold was 18.4 mm, tricipital skinfold 12.5 mm.

She was born from a pregnancy of 36 + 5 weeks characterized by gestational mellitus diabetes. Neonatal weight was 2900 g (AGA), length was 47.7 cm (AGA), head circumference was 33 cm (AGA) and Apgar score was 9/10. During the second day of birth, she developed severe hypoglycaemia that required glucose infusion. Her father and mother are not related, with proportional and normal height: mother height is 162 cm whereas father height is 180 cm (target height 164.5 cm). There are no cases of short stature in family members (only child). Parents’ origin was from North of Italy and Sicily.

Biochemically, blood count, creatinine, liver function, electrolytes, urine evaluation and serological screening for celiac disease were normal. Her serum basal GH levels were 11.57 ng/ml with glucose levels of 59 mg/dl, IGF-1 19.1 ng/ml (−2.7 SDS) and IGFBP-3 0.7 mcg/ml (2.0–4.5), respectively.

We failed to detect a positive response to the standard IGF-1 generation test by administering 0.033 mg/kg/day of rhGH for 4 consecutive days [IGF-1 d0: 13.6 ng/ml (−2.6 SDS); IGF-1 d4: 8.4 ng/ml (−2.8 SDS)], indicating a GH resistance.

**Fig. 2:**
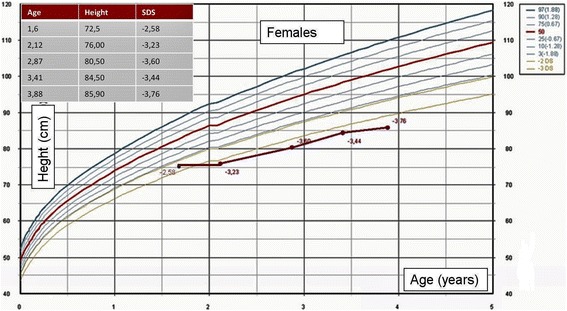
Auxological follow-up – Females height for age WHO 2006

Auxological follow-up for height is shown in Fig. 2.

At the age of 3.8 years old the height was 85.9 cm (−3.76 SDS), weight 11.9 kg (−2.42 SDS), head circumference 50 cm (0.52 SDS), and growth velocity deflected at 5.29 cm/year (−1.80 SDS). Basal IGF-1 levels were 32 ng/ml (−2.28 SDS) and IGFBP-3 0.8 mcg/ml (2.0–4.5). After a high dose of rhGH (2.8 mg/mq/die: 1.6 mg/die for 7 days), stimulated IGF-1 levels were 62.0 ng/ml (−1.78 SDS) and IGF1BP-3 0.9 mcg/ml (2.0–4.5). We decided to start rhIGF-1 (0.5 mg twice daily s.c.: 0.04 mg/kg/twice daily).

In the hypothesis of a case of Laron Syndrome, we performed the direct sequencing of the complete coding sequence of GHR gene that revealed the presence of two heterozygous variation. The first is a missense mutation caused by the transition adenine to guanine (c.1A > G) in the first codon of exon 2. Given that, this substitution involved the translation initiation codon (Methionine) of the protein, the correct expression of the receptor will be necessary inhibited. The second variation is the substitution c.307G > A, that resulted in the replacement of the amino acid Aspartic Acid at position 103 to a residue of Asparagine (p.D103N), in exon 5 of the GHR sequence. This substitution involved the highly conserved Aspartic Acid 103 and, as predicted by the PolyPhen-2 Program (http://genetics.bwh.harvard.edu/pph2), it could be responsible for a damaging effect on GHR functionality. Due to the identification of these variations in her daughter, the parents underwent a molecular analysis for GHR gene mutation. The direct sequencing demonstrated that the father was heterozygous for the first codon mutation, whereas the mother was instead heterozygous for the p.D103N variation.

## Discussion and conclusions

Heterozygous mutations in the GHR sequence have been frequently identified in children with idiopathic short stature, and these mutations should be taken into account when the other causes have been ruled out [11–13]. Laron Syndrome is a genetic disorder characterized by the inability to respond to endogenous or exogenous GH. This disorder is characterized by severe growth failure after birth, craniofacial disproportion, elevated serum GH and low/undetectable IGF-I levels that fail to respond to rhGH administration [3, 14].

In our patient, high doses of rhGH during IGF-1 generation tests demonstrated a partial response to IGF-1 production, that probably reflects the mild phenotype in her first years of life [15, 16]. After the third year, due to the failure of compensatory mechanisms, her growth velocity deflected and short stature should require IGF-1 substitutive therapy to exceed GH partial insensitivity [10].

Molecular analysis of the GHR gene demonstrated the presence of the novel variation p.D103N, which involved the highly conserved residue of Aspartic Acid at position 103 of the extracellular domain. It is well demonstrated that most of the mutations identified in children with short stature were localized in the extracellular domain of the receptor, and are responsible of an impaired GH binding [12]. Considering that, the hormone binding and the subsequent receptor dimerization, are essential steps to activate the GHR signalling transduction, we can hypothesized that our p.D103N variation is responsible of an impaired GH binding that causes an altered receptor functionality.

The other mutation identified in our patient involved the first ATG codon of the GHR sequence that encodes for the Methionine necessary for the translation of all the mature proteins. Because the first ATG is essential for the initiation of the translation, this mutation certainly inhibits the correct expression of the receptor. A similar mutation was previously described by Quinteiro and co-workers, [17] in a patient with Laron Syndrome. They observed that the successive ATG codon is located in the sixth exon and encodes for the methionine at position 188. Even if this mutation could be able to start the receptor translation, the resulting protein will be truncated by the first 187 amino acids and will probably be not functional.

It has been difficult to establish if the presence of a heterozygous mutation in the GH receptor can result in clinically significant GH insensitivity, except in the presence of a well-demonstrated dominant-negative effect. It can be assumed that, in patients who are heterozygous for a GHR mutation, the stature within the normal range may be achieved by a compensatory mechanism of GH secretion or signalling. On the contrary, homozygous or compound heterozygous patients would manifest a clinical GH insensitivity given that compensatory mechanisms are inadequate, and short stature may be the consequence [5].

## Abbreviations

GH: Growth Hormone
GHR: Growth Hormone Receptor
IGF-1: Insulin-like Growth Factor 1
IGFBP-3: Insulin-like Growth Factor Binding Protein 3
JAK-STAT: Janus chinasi - Signal Transducer and Activator of Transcription
SDS: standard deviation score
